# Identifying Kidney Stone Risk Factors Through Patient Experiences With a Large Language Model: Text Analysis and Empirical Study

**DOI:** 10.2196/66365

**Published:** 2025-05-22

**Authors:** Chao Mao, Jiaxuan Li, Patrick Cheong-Iao Pang, Quanjing Zhu, Rong Chen

**Affiliations:** 1 MPU-UC Joint Research Laboratory in Advanced Technologies for Smart Cities Faculty of Applied Sciences Macao Polytechnic University Macao Macao; 2 Department of Laboratory Medicine West China Hospital Sichuan University Chengdu China; 3 Department of Rehabilitation Medicine The First Affiliated Hospital Sun Yat-Sen University Guangzhou China

**Keywords:** health risk identification, chronic disease management, GPT-4, large language model, text analysis

## Abstract

**Background:**

Kidney stones, a prevalent urinary disease, pose significant health risks. Factors like insufficient water intake or a high-protein diet increase an individual’s susceptibility to the disease. Social media platforms can be a valuable avenue for users to share their experiences in managing these risk factors. Analyzing such patient-reported information can provide crucial insights into risk factors, potentially leading to improved quality of life for other patients.

**Objective:**

This study aims to develop a model KSrisk-GPT, based on a large language model (LLM) to identify potential kidney stone risk factors from web-based user experiences.

**Methods:**

This study collected data on the topic of kidney stones on Zhihu in the past 5 years and obtained 11,819 user comments. Experts organized the most common risk factors for kidney stones into six categories. Then, we use the least-to-most prompting in the chain-of-thought prompting to enable GPT-4.0 to think like an expert and ask GPT to identify risk factors from the comments. Metrics, including accuracy, precision, recall, and *F*_1_-score, were used to evaluate the performance of such a model.

**Results:**

Our proposed method outperforms other models in identifying comments containing risk factors with 95.9% accuracy and *F*_1_-score, with a precision of 95.6% and a recall of 96.2%. Out of the 863 comments identified with risk factors, our analysis showed the most mentioned risk factors for kidney stones in Zhihu user discussions, mainly including dietary habits (high protein, high calcium intake), insufficient water intake, genetic factors, and lifestyle. In addition, new potential risk factors were discovered with GPT, such as excessive use of supplements like vitamin C and calcium, laxatives, and hyperparathyroidism.

**Conclusions:**

Comments from social media users offer a new data source for disease prevention and understanding patient journeys. Our method not only sheds light on using LLMs to efficiently summarize risk factors from social media data but also on LLMs’ potential to identify new potential factors from the patient’s perspective.

## Introduction

Kidney stones are the most common urinary system disease, and the global incidence rate has been increasing in recent years. Given that kidney stones predominantly form in the renal calyx, renal pelvis, and ureteropelvic junction [1,2], an increase in stone size can result in clinical manifestations such as hematuria, nausea, dysuria, and acute pain in the lower abdomen or back. When kidney stones migrate from the kidneys into the urinary tract, patients may experience severe and debilitating pain. Therefore, many patients with kidney stones have already developed various clinical symptoms when they go to the hospital for treatment [3]. Since the detection process of kidney stones is long, there is not only a significant reduction in the patient’s quality of life but also a potential risk of renal function deterioration, which may ultimately threaten the patient’s life [4,5]. Therefore, early prevention of kidney stones is of great significance.

The incidence of kidney stones varies depending on gender, age, ethnic background, geographic location, and dietary differences. Hypercalciuria, hyperoxaluria, obesity, diabetes, insulin resistance, and other related diseases are common risk factors for stone formation [6-8]. Despite these recognized risks, patients still have low awareness of kidney disease and its potential safety risks and lack communication with doctors, which greatly increases the risk of disease [9-11]. Therefore, identifying the risk factors associated with kidney stones can serve as a valuable reference for individuals, enabling them to implement preventive measures in their daily lives to reduce the incidence of this condition.

Previously, many studies have been conducted to identify risk factors for kidney stones. The investigations in question gathered participants from particular age brackets or geographical regions, using surveys to amass data on demographics, lifestyle patterns, familial medical backgrounds, coexisting health conditions, and residential locations. Following this, the researchers used relevant analytical techniques to examine the correlations between these collected data points and the occurrence of kidney stones. The ultimate goal was to pinpoint potential risk factors for kidney stone formation within these specific demographic or geographic subsets [12-14]. Nevertheless, these investigations are not without their constraints. Questionnaires are limited in their ability to gather data on specific information, resulting in the identification of incomplete and constrained risk factors, offering minimal insight for the broader patient population. Additionally, these studies demand prolonged investigations and significant effort to gather meaningful feedback. Conversely, collecting information from social media platforms is more cost-effective and can encompass a larger population [15]. On social media platforms, users can share their opinions and experiences on a variety of topics [16,17] and even share their personal experiences of illness [18]. Over time, highly unstructured tacit knowledge is generated in communities where users frequently participate [19,20]. Moreover, users on social media platforms span different ages, genders, and regions, making the information more comprehensive and representative. In summary, content on social media can be used as a data source for identifying risk factors and understanding the challenges faced by patients.

Text mining techniques are generally used to process social media data [21,22]. Some researchers have sought to gain knowledge about disease risk factors from social media data [23,24]. However, these studies only used shallow textual features in the data. For texts in which users share their experiences of illness, it is difficult to identify disease risk factors due to the colloquial and diverse nature of the texts [25]. Recent years have seen a substantial improvement in text data processing skills because of the quick development of artificial intelligence technologies, particularly with the rise of large language models (LLMs). LLMs are natural language processing (NLP) models built on the transformer architecture. After training with an enormous quantity of text data, it can understand and generate text like human language [26]. LLMs perform well in medical text tasks and can conduct in-depth analyses of large amounts of unstructured medical text, improving the efficiency of medical data processing and the accuracy of clinical decision-making [27]. In addition, LLMs are also good at processing social media data and can identify potential disease risk factors from the experiences and opinions shared by users [28]. Prompt engineering, as an emerging technology, guides LLMs to generate more accurate and relevant content by designing specific prompt words or sentences, thereby improving the output quality and interpretability of the model [29]. This will contribute to better identifying risk factors for kidney stones, promoting early prevention and intervention, and enhancing the quality of life and overall health of patients.

This study aims to explore the disease risk factors contained in the disease experience texts shared by patients with kidney stone on social media platforms. We developed an innovative risk factor identification method named KSrisk-GPT based on GPT-4.0. This innovative approach aims to address the limitations of traditional classification models. Previous models often rely on surface text features such as word frequency, resulting in information loss and a lack of precision. Considering that social media users often mention related topics such as symptoms and bad habits when describing risk factors, the introduction of LLMs can more comprehensively understand and analyze the semantic content in the text [30].

Furthermore, we adopted the least-to-most (LtM) prompting engineering technology [31], which can help LLMs deeply explore the topic information of each risk factor to improve the recognition accuracy of specific risk factor phrases. By systematically identifying and analyzing risk factors from texts shared by patients on their own initiative, we provide new insights into the early prevention of kidney stones. The application potential of this method can also be extended to other chronic diseases, providing valuable data support for disease prevention and public health policy making.

In sum, this paper presents an LLM-based model (KSrisk-GPT) to identify the risk factors of kidney stones from patient-generated content about their experiences. Additionally, this work reports on the risk factors identified with this approach, which shows that our approach can identify risk factors from existing patient-generated content.

## Methods

### Framework

**Figure 1:**
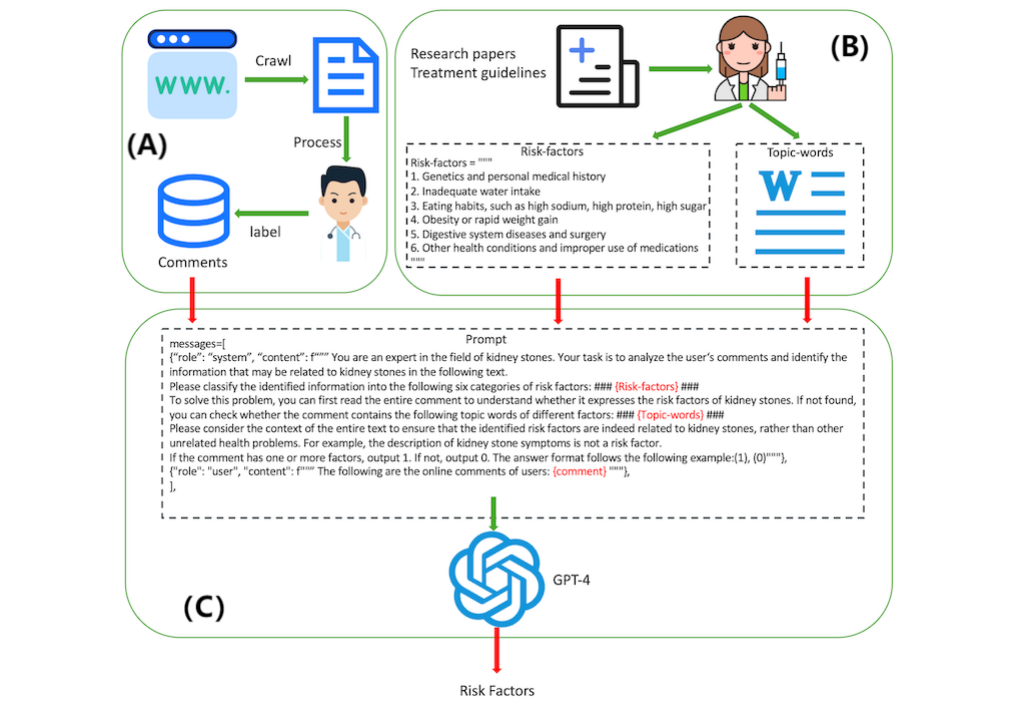
A method for identifying kidney stone risk factors based on GPT-4. (A) Data collection and processing, aiming to obtain a clean dataset. (B) Professional doctors participate in the construction of LLMs, summarize the risk factors of kidney stones, and organize related topic words through the latest relevant literature and medical guidelines. (C) Identification of risk factors, using the LtM prompt engineering method and combining the above-mentioned Risk-factors and Topic-words to identify risk factors in user comments. LLM: large language model; LtM: least-to-most.

The framework used in this study is composed of three parts, as illustrated in Figure 1. The first part involves data collection and processing. The data crawled from social media needs to be preprocessed and then screened and labeled by two professional doctors to obtain a clean dataset. The second part is to involve professional doctors in the construction of LLMs, aiming to improve the accuracy of LLMs and enhance the trust of others in the method. Doctors classified the risk factors of kidney stones into 6 categories through the latest relevant literature and medical guidelines, and sorted the subject words related to these 6 categories of risk factors into Topic-words. The third part is the identification of risk factors. The LtM prompt engineering method and the abovementioned Risk-factors and Topic-words can be used to identify risk factors in user comments.

Given that this study focuses on the analysis of Chinese user comments, we systematically evaluated the most popular large Chinese language models currently available before selecting the base model, specifically including versions 4.0 and 3.5 of ERNIE Bot, as well as the Kimi large model [32]. During the evaluation process, consistent prompt sentences were used, and 100 randomly selected comments related to risk factors for kidney stones were used as test samples. The experimental results revealed that the accuracy rate of ERNIE Bot version 4.0 was 82%, while that of version 3.5 was 75%, and the accuracy rate of the Kimi large model was 72%. These results did not reach the level demonstrated by the GPT series of models. It is noteworthy that the ChatGPT model launched by OpenAI has demonstrated significant advantages and broad development potential across multiple domains [33,34]. As a multilingual processing model, ChatGPT can efficiently analyze information in various languages, including Chinese. Particularly in innovative applications within the health care field, ChatGPT’s performance stands out prominently, surpassing all the aforementioned Chinese large models. Furthermore, given that ChatGPT has been widely adopted and extensively studied, it possesses high versatility and comparability, providing a rich reference framework and solid support foundation for our subsequent research work. Therefore, based on the above analysis, we have decided to adopt ChatGPT as the core model for this study. Recent research indicates that GPT-4.0, the latest version of ChatGPT, outperforms GPT-3.5 in several medical tasks [35,36]. Compared to GPT-3.5, GPT-4.0 has been shown to have improved capabilities in understanding complex medical terminology, reasoning through complex medical scenarios, and generating more coherent and contextually appropriate responses. These advances make GPT-4.0 a more suitable choice for our approach.

### Dataset

Zhihu is a well-known Chinese social media platform similar to Quora in the United States. It mainly provides social networking and question-and-answer services [37]. As of December 2020, the total number of questions on Zhihu exceeded 44 million, and the total number of answers exceeded 240 million. As one of the largest user-generated content platforms in China, Zhihu gathers a large number of text answers from various groups, which is considered an extensive and comprehensive data source for academic research [38]. On Zhihu, users can discuss various topics and comment on posts [39], including health care. By sharing their personal experience, patients can effectively provide support or seek help on the platform, therefore, Zhihu is a representative and valuable data source for studying various user-reported risk factors, as indicated in other research [40].

### Data Preprocessing

Zhihu organizes posts and comments on common topics in subcommunities. We searched all subcommunities with the keyword “kidney stone,” and collected all comments in such subcommunities in the past 5 years. This data collection step obtained a total of 11,819 comments. Before processing these data, we performed a preliminary data cleaning to remove comments that did not contain substantive content, such as “Thank you very much!” Subsequently, we used regular expressions to effectively remove special characters in the comments, including URL links and emoticons, to reduce the noise interference that may be generated in the subsequent text analysis process and improve the data quality. It is worth noting that although Zhihu’s data is publicly accessible and many studies have been conducted based on Zhihu’s comments, we adhere to the principles of respecting users and protecting personal privacy when processing this public data. For all comments that may contain personal information, we have taken strict manual filtering measures to ensure the compliance of data use. All remaining comments after data preprocessing were fed into GPT to identify risk factors.

Data in social media generally has a certain degree of ambiguity and tends to contain more colloquial expressions. Under the framework of traditional text processing technology, such data often needs to go through a more complicated preprocessing stage to lay the foundation for subsequent analysis. In some cases, these ambiguous data may even be directly filtered out. However, with its accumulation of large-scale pretraining datasets, GPT has demonstrated satisfactory contextual understanding and semantic analysis capabilities. This feature enables GPT to efficiently deal with slang, spelling errors, and unclear expressions [41-43]. When processing comment data in social media, GPT can automatically detect and correct typos and accurately identify the user’s true intentions and expressions. For example, when faced with ambiguous expressions such as “My dad got it the year before last,” the GPT model can combine contextual information to reasonably infer the specific situation of “My dad has kidney stones,” and then identify the key risk factor of the user’s possible family history. The use of such technologies has greatly improved our ability to quickly and accurately identify and extract useful information from massive social media data, providing strong support for users’ health monitoring work.

After preprocessing, the two experts (QZ and RC) labeled each comment. They based their annotations on a combination of personal experience and the latest medical literature to ensure the accuracy of the review labels. To ensure label consistency, all reviews were labeled by two experts (QZ and RC). If there was uncertainty in the label judgment, the two experts discussed and finally reached a consensus on the label. Finally, we obtained 863 comments identified with risk factors, and then randomly selected the same number of comments from the remaining comments that did not contain risk factors to form the experimental dataset for this study. Table 1 shows some examples of user comments with and without risk factors.

**Table 1:** Comment examples translated from Chinese^a^.

Comment	Label	Risk factors
1. My dad has kidney stones; I don’t like to drink water.	1 (risk factor)	Genetics, lack of water
2. The reason why I have an increased risk of stones is that I did not give up eating greasy meat, eggs, milk, fish, and shrimp	1 (risk factor)	Eating habits
3. I’m in so much pain, how can I get rid of it?	0 (non–risk factor)	No risk factor

^a^A comment may contain one or more risk factors. For example, comment 1 contains two risk factors and comment 2 contains only one risk factor. It is worth noting that some comments are descriptions of kidney stones but do not include risk factors, as indicated in comment 3.

### The Role of Medical Experts

To enhance the model’s transparency and precision, as well as increase health care practitioners’ confidence in the approach, this paper fully included the participation of medical experts in the method design [44]. First, in the data collection and processing stage, we crawled a large number of user comments about kidney stones from social media platforms. These raw data usually contain a lot of noise and irrelevant information, so strict preprocessing is required. To ensure the reliability and medical relevance of the data, we invited two senior medical experts (QZ and RC) to participate in the screening and labeling of the data. This rigorous process not only keeps the dataset clean but also enhances its medical professionalism. Second, medical experts refer to authoritative medical literature and guidelines such as the American College of Physicians Clinical Practice Guidelines and the Canadian Urological Association Guidelines: Evaluation and Medical Management of Patients with Kidney Stones and systematically classify the risk factors for kidney stones into 6 categories [45,46]. Based on these 6 categories of risk factors, experts further sorted out relevant topic words and saved them in Topic-words, which cover various possible expressions related to risk factors. This provides a professional and authoritative knowledge framework for LLMs and ensures that the model can be professional and accurate when processing information related to kidney stones. Finally, in the risk factor identification stage, we applied LtM prompting, combined with the risk factors and topic words sorted by the above experts, to analyze and identify user comments. Specifically, by embedding these keywords and risk factors in the prompt engineering, the model can more accurately identify potential kidney stone risk factors in user comments.

In summary, through the in-depth participation of medical experts in every aspect, from data preprocessing to model construction to risk factor identification, our method has been significantly improved in terms of accuracy and interpretability. This cross-field cooperation model not only provides a solid medical foundation for our research but also provides new ideas for the development of future medical artificial intelligence models.

### LtM Prompting

Prompt engineering is an emerging research field that focuses on the careful design, optimization, and application of prompts and instructions. These prompts and instructions are designed to guide LLMs to produce specific output to efficiently complete a variety of complex tasks [29]. In our experiments, the prompt language used is Chinese, which places higher requirements on LLMs. In order to effectively cope with the processing requirements of Chinese data, we specially combined the language characteristics and context of Chinese when designing prompts. We implemented a progressive prompt engineering approach, which involves transitioning from minimal to more extensive prompts in a step-by-step process [31]. This process aims to gradually improve the model’s recognition accuracy and robustness of kidney stone risk factors. Our LtM prompting process includes the following steps.

As shown in the Multimedia Appendix 1, we provide KSrisk-GPT with a simple initial prompt, asking it to identify content in the comments that may be related to kidney stones. Next, we introduce 6 categories of kidney stone risk factors defined by medical experts and ask the model to classify the identified information. In this step, we incorporate Topic-words sorted by experts and allow the model to explore associations beyond these keywords, enabling more accurate classification of previously uncertain comments. Finally, the model is required to consider the context of the text to ensure that the identified risk factors are genuinely related to kidney stones, rather than other unrelated health issues, such as the description of kidney stone symptoms as not a risk factor.

Through this LtM prompting process, we gradually guide GPT-4 to deeply analyze the text, thereby improving the accuracy and reliability of identifying kidney stone risk factors. This approach not only uses the expertise provided by doctors but also fully uses the advantages of LLMs in language understanding and context analysis.

### Experiment

Text classification is a common task in NLP, and research has already been conducted on classifying patient comments [47]. In addition, text classification technology is constantly improving. The transformer architecture proposed in 2017 solves the long-distance dependency problem through the self-attention mechanism [48] and has become the basis for many subsequent breakthrough models. Among them, Bidirectional Encoder Representations from Transformers (BERT) [49] has created new benchmarks in multiple NLP tasks through bidirectional encoding and pretraining-fine-tuning paradigms. Recently, LLMs represented by the GPT series have demonstrated powerful learning capabilities, further promoting the development of text classification technology. To highlight the advantages of KSrisk-GPT in social media text recognition, we introduced three text classification models for comparison: Text-CNN (convolutional neural network), BERT, and Transformer. The comparative experiment required splitting the dataset into a training set to train the model and a validation set to verify the results. In our experiments with the KSrisk-GPT model, to efficiently evaluate a large number of comments, we developed an automated script that uses the GPT-4.0 interface provided by OpenAI. This method avoids manual input, ensuring consistency and objectivity. Our process includes data preparation, automated processing, concurrency optimization, and results collection with metric calculations.

### Comparative Experimental Design

In this study, we also used supervised learning to train three other models, Text-CNN, BERT, and Transformer, to ensure the fairness and comparability of the experimental results. The hyperparameters of all models were kept consistent. We first preprocessed the text data, including removing extra spaces and punctuation marks, and used the Jieba word segmentation tool to segment the text, while removing stop words. Then, the word2vec method was used to generate 100D word vectors as the input of the model. Specifically, we set the learning rate to 0.0001, the batch size to 32, and trained for 30 rounds. To evaluate the performance of the model, we randomly split the dataset into an 80% training set and a 20% test set. This split ensures the independence between the training set and the test set so that the generalization ability of the model can be more accurately evaluated. During the training process, we recorded the performance of each model on the test set and conducted a detailed analysis and comparison. Through these steps, we successfully trained and evaluated three models to explore their performance on specific tasks.

### Evaluation Metrics

This study used a number of evaluation measures, such as accuracy, precision, recall, and *F*_1_-score, to evaluate the prediction model’s performance. Accuracy offers a broad overview of the model’s performance and indicates how well the model predicts things overall. Precision measures how accurate the model is when it makes a positive judgment and is calculated as the ratio of correct positive predictions to all positive predictions. Recall reflects how comprehensively the model identifies relevant cases and is calculated by dividing the number of correctly identified relevant instances by the total number of all actual relevant instances. The *F*_1_-score, as the harmonic mean of precision and recall, reflects the model’s balance between classification reliability and completeness. Together, these metrics form a comprehensive evaluation system that helps to gain a deeper understanding of the performance characteristics of the model.

### Ethics Approval

This study has obtained a human ethics approval from the Faculty of Applied Sciences of Macao Polytechnic University (project ID: HEA001-FCA-2024).

## Results

This study compares the performance of 4 models on the social media text risk identification task: KSrisk-GPT, Text-CNN, BERT, and Transformer. As the experimental results in Table 2 show, KSrisk-GPT is significantly better than the other three models in all evaluation indicators, reaching 95.9% accuracy and *F*_1_-score, while the performance indicators of other models are mostly between 80% and 85%. Among traditional models, Transformer performed best overall (84% accuracy), followed by Text-CNN (83.6% accuracy), and BERT’s performance was slightly lower (80.7% accuracy). The excellent performance of KSrisk-GPT is attributed to its large-scale pretraining base, domain adaptability, and strong context-understanding capabilities. Although the performance of traditional models is not as good as KSrisk-GPT, they still show good recognition capabilities with low computing resource requirements and may have more advantages in some practical application scenarios. Overall, the experimental results show that methods based on large-scale language models have significant advantages in social media text risk identification tasks, providing new directions for the development of related applications in the future.

**Table 2:** Experimental results for each model.

Model	Precision (%)		Recall (%)			*F*_1_-score (%)			Accuracy (%)
	Label^a^ 0	Label 1	Label 0	Label 1	Label 0		Label 1		
KSrisk-GPT (Ours)	91.2	95.6	95.6	96.2	95.9		95.9	95.9	
Text-CNN^b^	81	86.4	86.5	80.9	83.6		83.6	83.6	
BERT^c^	78.1	83.5	83.7	77.9	80.8		80.6	80.7	
Transformer	80	88.7	89.2	79	84.4		83.6	84	

^a^Label 0 indicates nonrisk factors and 1 indicates risk factors.

^b^CNN: convolutional neural network.

^c^BERT: Bidirectional Encoder Representations from Transformers.

This study analyzed 863 comments containing risk factors for kidney stones, revealing a variety of key factors that affect kidney stone formation. Table 3 shows the distribution of the categories of risk factors. Genetics and personal medical history were the most mentioned risk factors (n=994, 57.3%), indicating that people with a genetic predisposition to kidney stones and those who have previously experienced them face an increased likelihood of developing stones again. Insufficient water consumption ranked as the second most prevalent risk element (n=994, 22.6%), highlighting the crucial importance of proper water intake in kidney stone prevention. Low water consumption can result in urine concentration and elevate the likelihood of mineral accumulation. Dietary habits (n=994, 7.8%) were also considered important factors, including high sodium, high protein, and high sugar diets, which may increase the risk of stone formation by affecting the balance of urine components. Other health conditions and medication use (n=994, 9.8%) also play an important role in kidney stone formation. This includes specific health problems (such as renal tubular acidosis, cystinuria, and hyperparathyroidism) and improper use of certain medications and supplements (such as vitamin C, laxatives, and calcium supplements). Digestive diseases and surgery (n=994, 1.5%) may increase the risk of stones by affecting calcium and water absorption, while obesity (n=994, 0.9%) may promote stone formation by changing the balance of chemicals in the body.

**Table 3:** Distribution of risk factors where some reviews included one or more risk factors.

Risk factors	Count	Percentage
Genetics or personal medical history	570	57.3
Lack of water	225	22.6
Eating habits	78	7.8
Obesity	9	0.9
Digestive system diseases and surgeries	15	1.5
Other health conditions or excessive intake of supplements	97	9.8

It should be noted that the aggregate count of references (n=994) surpassed the quantity of remarks (n=863), suggesting that numerous instances encompass several risk factors, underscoring the intricacy and multifaceted character of kidney stone development. These findings not only emphasize the importance of genetics, personal medical history, and lifestyle (especially water intake and dietary habits) in kidney stone prevention but also provide an important basis for the development of targeted prevention strategies and public health education. Although some factors, such as obesity and digestive system problems, were mentioned less frequently in this study, they still need to be given sufficient attention in future studies, considering their potential impact on kidney stone formation. Based on these findings, it can be concluded that the prevention and management of kidney stones requires comprehensive consideration of multiple risk factors and personalized intervention measures.

To acquire a more profound insight into the linguistic characteristics of discussions related to kidney stones, we conducted word frequency statistics on comments with and without risk factors and generated corresponding word cloud diagrams (Multimedia Appendix 2). This visualization method not only visually displays the most used words but also reveals the differences in the language used by the two groups of comments. Word frequency analysis showed that in comments containing risk factors, words such as “number of times,” “years,” and “drink water” that are related to known risk factors appeared more frequently, which is consistent with our previous risk factor analysis results. In contrast, comments without risk factors may contain more words related to symptom description, diagnostic process, or treatment methods. The word cloud further highlights these differences, allowing us to observe the focus of the two groups of comments immediately. It is worth noting that some common words (such as “kidney stones” and “pain”) appear frequently in both groups of comments, reflecting the universality of these terms in discussions related to kidney stones. This word frequency analysis and word cloud visualization not only helps to verify our previous identification of risk factors but also provides a new perspective for understanding the public’s awareness and concerns about kidney stones.

## Discussion

### Principal Findings

Our proposed model can identify risk factors for kidney stones, such as dietary habits (high protein and high calcium intake), insufficient water intake, genetic factors, and lifestyle, with satisfactory performance. Our approach can also reveal novel potential risk factors, including excessive use of supplements like vitamin C and calcium, laxatives, and hyperparathyroidism, highlighting the value of unstructured data from patients in enhancing medical insights.

Compared to traditional clinical consultations, this method can capture behaviors and habits that are difficult to detect, supporting doctors to pay more attention to these factors when diagnosing and formulating treatment plans [50]. The current methods for identifying disease risk factors on social media platforms are mainly divided into shallow text feature methods and discrete word vector representations. However, both methods have certain limitations in practical applications [51-54]. Shallow text feature techniques perform poorly in capturing key risk factors, resulting in low accuracy. Discrete word vector methods are unable to cope with the dynamic nature and linguistic diversity of the text, making it difficult to effectively track emerging vocabulary and expression trends, hence failing to comprehensively and accurately represent the semantic information contained in the text. Our proposed KSrisk-GPT model can significantly improve the identification effect of disease risk factors, can better capture the semantic information and contextual relationships of text, and can flexibly respond to dynamic changes in language [55]. This allows for more efficient processing and analysis of unstructured data provided by patients, such as web-based medical histories. This capability greatly reduces the burden on doctors when processing large amounts of information, allowing them to focus more on key aspects of clinical decision-making.

The method used in this study enables doctors to be more proactive in patient health management [56]. Doctors can develop more personalized treatment plans by tracking the living habits and health status shared by patients. For example, certain eating habits or lifestyles may be the main cause of kidney stones in a particular patient. Based on this data, more accurate treatment advice and life guidance can be provided. At the same time, we also found some new potential risk factors, such as overuse of certain health supplements and abnormally high parathyroid hormone levels. In Zhihu user discussions, many users who took health supplements for a long time and in large quantities reported the appearance of kidney stones. Studies in the literature [57,58] have shown that excessive intake of vitamin D may lead to increased blood calcium levels, thereby promoting the formation of kidney stones. Another study found that long-term and large intake of fish oil may interfere with the metabolism of certain components in urine and increase the risk of kidney stones. Therefore, the results of this study echo the conclusions of existing studies, further emphasizing the need for caution when using health supplements and following the advice of doctors. In addition, high parathyroid hormone levels are also an important new risk factor. High parathyroid hormone levels can lead to increased blood calcium levels, thereby increasing the risk of kidney stones. However, most existing studies have focused on patient groups with primary or secondary high parathyroid hormone levels. This study also found high parathyroid hormone levels in ordinary users, which may be related to diet, lifestyle, and certain health conditions. This finding not only provides a new perspective on the risk factors for kidney stones but also suggests that we should pay more attention to the problem of high parathyroid hormone levels in the general population and conduct more in-depth investigations and research. In summary, doctors can use this method to detect earlier that certain patients may be exposed to potential risk factors, and to intervene in advance. This reduces not only the incidence of disease but also the medical costs and the waste of resources.

Although our current research focuses on kidney stones, the proposed model also has broad application potential in other health-related areas. For example, in asthma research, existing studies [59] have used keywords in existing knowledge bases to extract behavioral features from Twitter posts of asthma users. Our model can combine the annotations of medical experts and existing knowledge bases to extract behavioral features of patients with asthma from social media data, further enriching the understanding of their lifestyle and behavioral habits. In diabetes research [51], researchers collected 25,000 tweets containing and not containing diabetes, identified 5000 common words, and used logistic regression and latent Dirichlet allocation methods to identify and group risk factors for diabetes. Our model, with the advantage of LLMs, can identify more complex information, such as slang, thereby improving its application value in diabetes research. In addition, some researchers have used syntactic analysis methods to extract risk factors for gastrointestinal diseases from tweets, combining data from social media and medical literature [52]. Our model can also effectively extract these risk factors with a deeper understanding of the context. In line with recent research [60,61], the rapid development of LLMs can be used in analyzing patient experience data, which is a valuable tool for conducting patient-centric research.

### Limitations

While this study offers a significant understanding of the trends in the perception of kidney stone risk factors, there are limitations worth noting. First, our analysis was mainly based on self-reported information from users, which lacks verification by medical professionals and may have issues with information accuracy and reliability. Second, this study failed to quantify the relative importance of individual risk factors or their interactions, which may pose a challenge to a comprehensive understanding of kidney stone risk factors.

### Conclusions

This study has successfully extracted and analyzed potential risk factors for kidney stones from social media data using an innovative approach called KSrisk-GPT, which is based on LLMs. Our work has identified the well-known major risk factors, such as poor eating habits and insufficient water intake, and has also uncovered some new potential risk factors that can help people avoid these health issues. With the rapid advancement of LLMs, our research underscores the practicality and feasibility of using LLMs to analyze patient-reported data for risk factor research. This sheds light on the potential adoption of LLMs in future public health research.

## Appendix

Multimedia Appendix 1 Prompts.

Multimedia Appendix 2 Word clouds of (A) reviews without any risk factors and (B) reviews with risk factors.

## Abbreviations

BERT: Bidirectional Encoder Representations from Transformers
CNN: convolutional neural network
LLM: large language model
LtM: least-to-most
NLP: natural language processing

## Data Availability

The datasets generated during or analyzed during this study are available from the corresponding author upon reasonable request.
