# Supplementary material for: Identifying Kidney Stone Risk Factors Through Patient Experiences With a Large Language Model: Text Analysis and Empirical Study
Source: J Med Internet Res. 2025 May 22;27:e66365. doi: 10.2196/66365 (PMC12141965; doi:10.2196/66365)
Supplement: Multimedia Appendix 1 [file jmir_v27i1e66365_app1.docx]

Prompt：

messages = [

{"role": "system", "content": f""" You are an expert in the field of kidney stones. Your task is to analyze the user's comments and identify the information that may be related to kidney stones in the following text.

Please classify the identified information into the following six categories of risk factors: ### {Risk-factors} ###

To solve this problem, you can first read the entire comment to understand whether it expresses the risk factors of kidney stones. If not found, you can check whether the comment contains the following topic words of different factors: ### {Topic-words} ###

Please consider the context of the entire text to ensure that the identified risk factors are indeed related to kidney stones, rather than other unrelated health problems. For example, the description of kidney stone symptoms is not a risk factor.

If the comment has one or more factors, output 1. If not, output 0. The answer format follows the following example:(1), (0)"""},

{"role": "user", "content": f""" The following are the online comments of users: {comment} """},

]

where:

Risk-factors = """

1. Genetics and personal medical history

2. Inadequate water intake

3. Eating habits, such as high sodium, high protein, high sugar

4. Obesity or rapid weight gain

5. Digestive system diseases and surgery

6. Other health conditions and improper use of medications

"""
