# Supplementary figures and images for: Identifying Kidney Stone Risk Factors Through Patient Experiences With a Large Language Model: Text Analysis and Empirical Study
Source: J Med Internet Res. 2025 May 22;27:e66365. doi: 10.2196/66365 (PMC12141965; doi:10.2196/66365)

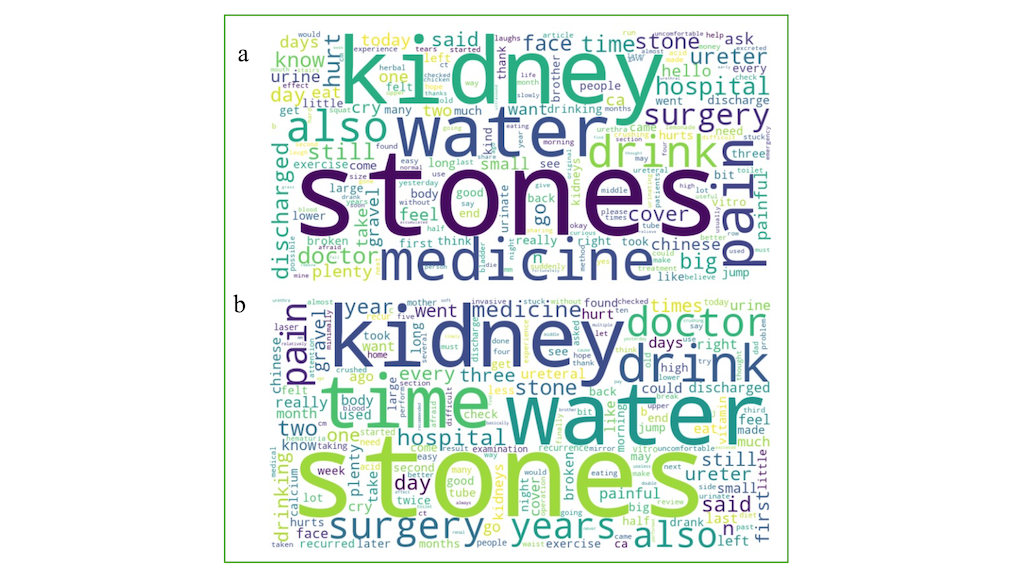

Supplement: Multimedia Appendix 2 [file jmir_v27i1e66365_app2.png]
